# Feasibility of Training a Random Forest Model With Incomplete User-Specific Data for Devising a Control Strategy for Active Biomimetic Ankle

**DOI:** 10.3389/fbioe.2020.00855

**Published:** 2020-08-07

**Authors:** Sharmita Dey, Takashi Yoshida, Arndt F. Schilling

**Affiliations:** Applied Rehabilitation Technology Lab (ART-Lab), Department of Trauma Surgery, Orthopedics and Plastic Surgery, University Medical Center Göttingen, Göttingen, Germany

**Keywords:** human gait, prediction, prosthetic control, intelligent biomimetics, random forest

## Abstract

Intelligent control strategies for active biomimetic prostheses could exploit the inter-joint coordination of limbs in human gait in order to mimic the functioning of a biological joint. A machine learning regression model could be employed to learn an input-output relationship between the coordinated limb motion in human gait and predict the motion of a particular limb/joint given the motion of other limbs/joints. Such a model could be potentially used as a controller for an intelligent prosthesis which aims to restore the functioning similar to an intact biological joint. For this, the model needs to be tailored for each user by learning the gait pattern specific to the user. The challenge of training such machine learning regression models in prosthetic control is that, the desired reference output cannot be obtained from an amputee due to the missing limb. In this study, we investigate the feasibility of using two different methods for training a random forest algorithm using incomplete amputee-specific data to predict the ankle kinematics and dynamics from hip, knee, and shank kinematics. First is an inter-subject approach which learns a generalized input-output relationship from a group of able-bodied individuals and then applies this generalized relationship to amputees. Second is a subject-specific approach which maps the amputee's inputs to a desired normative reference output calculated from able-bodied individuals. The subject-specific model outperformed the inter-subject model in predicting the ankle angle and moment in most cases and can be potentially used for devising a control strategy for an intelligent biomimetic ankle.

## 1. Introduction

Lower limb amputation hinders the quality of life. The passive replacements of a missing limb are incapable of restoring normal gait (Varol and Goldfarb, 2007; Windrich et al., 2016). Active or intelligent biomimetic prostheses that are actuated using embedded motors can better support natural gait by substituting the missing muscle function and providing adequate torque (Varol and Goldfarb, 2007; Windrich et al., 2016). The central unit of such active prostheses is an intelligent control strategy that interprets the user's locomotive intention from the residual limb motion (Villarreal et al., 2016; Bartlett and Goldfarb, 2017) and thereby actuate the prosthetic joints to enable the desired locomotion similar to that of able-bodied individuals'. To define appropriate control strategy for such active prostheses, the functioning of a biological joint during human gait can be mimicked. A machine learning regression model could be employed to learn an input-output relationship between the coordinated limb motion in human gait (Boudali et al., 2017) and predict the motion of a particular limb/joint given the motion of other limbs/joints. Such a model could be potentially used as a controller for an intelligent prosthesis which aims to restore the functioning similar to an intact biological joint.

The development of a control strategy for active prostheses can be achieved in four stages: (1) selecting the control model, (2) selecting model input features, (3) selecting model outputs, and (4) establishing an input-output relationship.

The first stage is choosing the control model that translates the user's locomotive intention inferred from sensory input to output commands for actuating the active prosthetic joints. Existing control models can be broadly classified as discrete or continuous. In the discrete approach, a gait cycle (or a stride) is divided into discrete phases and inputs from external sensors are used to recognize the intended gait phase of a particular locomotion mode (Sup et al., 2008; Varol et al., 2010). For each kinematic or kinetic variable (i.e., state) to be controlled, its desirable value is specified by a finite-state controller, which uses a lookup table that corresponds to the recognized gait phase. A finer division of the gait cycle as well as an increase in the number of locomotion modes or states to be controlled result in an exponential increase in the number of parameters to be tuned as well as look-up tables to be maintained (Lawson et al., 2013; Tucker et al., 2015). On the other hand, in a continuous approach, the prosthesis kinematics or kinetics (state) is continuously varied depending on the sensor input. Machine learning regression algorithms have recently received increased attention as a continuous control model for active prostheses (Eslamy and Schilling, 2018; Dey et al., 2019). Unlike the discrete finite-state models, machine learning regression models can learn a continuous relationship between the sensor input and the output control variables (prosthesis kinetics or kinematics) to be predicted.

The second stage is choosing the input to the control model. The input should reflect the user's locomotive intention and enable the model to learn a robust input-output relationship for a given prosthesis user. Some of the feasible input choices are residual limb kinematics (Holgate et al., 2009; Quintero et al., 2016) or muscle activation (Au et al., 2005; Huang et al., 2011).

The third stage is choosing the outputs of the control model. The choice of the outputs depends on what best governs locomotion in specific scenarios [e.g., type of prosthesis, kinematic and kinetic behaviors of the prosthetic joints, balance, or efficiency of gait (Jezernik et al., 2003; Au et al., 2008; Eilenberg et al., 2010; Tsukahara et al., 2011)].

The fourth stage is establishing an appropriate relationship between the selected input features and outputs, such that the control model can realize the desired locomotion. This stage is particularly critical to ensure adequate prosthetic control and is the main focus of our study. For a machine learning regression model, the aforementioned input-output relationship is established through training. The challenge of training the machine learning regression models for active prosthesis control is that the complete training data cannot be obtained from amputees due to the missing limb. One way to address this problem is by using an inter-subject approach that seeks a generalized input-output relationship using locomotion data from a group of able-bodied individuals (Ardestani et al., 2014) and is applied to the prosthetic user. The advantage of the inter-subject approach is that, once the generalized input-output relationship is established, the model requires no training data to be collected from the new individual. However, an obvious disadvantage is that the prediction accuracy may suffer if the input from a particular individual substantially deviates from the training data due to inter-individual variations in gait (Hof, 1996; Stansfield et al., 2003; Senden et al., 2012; Wahid et al., 2016; Allard et al., 2017). This is especially relevant for prosthetic control, as amputees are more likely to exhibit gait abnormalities (Winter and Sienko, 1988; Silverman et al., 2008). While some studies suggest to account for the inter-individual variability in gait by scaling the input features (Pinzone et al., 2016; Allard et al., 2017), the parameters for scaling are typically anthropometric and likely not effective in compensating for the inherently different gait patterns across individuals.

Another way to address this could be a subject-specific approach that identifies a unique input-output relationship based on the experimental data from a particular individual (Dey et al., 2019). However, a subject-specific approach cannot be directly applied for amputees due to the absence of desired reference outputs from the amputee her/himself. Hence, to make the subject-specific model applicable for amputees, we used a modified subject-specific approach to learn a relationship between the amputee's input and a normative reference output calculated from a sample of able-bodied subjects' walking trials at a similar speed as that of the amputee. The main advantage of this approach is that the input from a specific user need not be similar to that of a group of able-bodied individuals to produce the desired normative output values.

In this study, we assessed the feasibility of training a random forest model with incomplete user-specific data for designing a potential control strategy for an active biomimetic ankle prosthesis. To achieve this, we compared the two types of models: inter-subject and the subject-specific, to continuously predict the ankle angles and moments within gait cycles based on random forest regression (Breiman, 2001). We chose random forest regression for devising the control model because it possesses characteristics that make it more suitable than other competing algorithms for controlling an active prosthesis. First, random forest has demonstrated to give a more robust and accurate prediction performance compared to other algorithms like Gaussian process regression and support vector regression (Hultquist et al., 2014). Unlike other machine learning models (e.g., the distance-based models), where it is recommended that the features be normalized to attain a high prediction accuracy or a faster convergence (Hsu et al., 2003; Khah and Wu, 2019), random forests do not require input feature normalization, which makes it more suitable for real-time usage. Additionally, random forests can achieve high prediction accuracy being trained on a small training set and with less training time as opposed to many other machine learning algorithms (Douglas et al., 2011; Biau, 2012; Biau and Scornet, 2016). As a result, only a minimal amount of data might be required from the amputee for training the prosthesis controller. Moreover, random forests are easily parallelizable and hence suitable for dealing with a large amount of training data in real-time (Biau and Scornet, 2016). This can come to benefit when updating/learning the model online, to continuously adapt to new training data over time, in order to account for changes in locomotion speeds, patterns, or the prosthetic setup. Lastly, random forests can quantify the relative importance of input features in decision making, which can be used for feature selection and dimensionality reduction.

The hip, knee, and shank kinematics were used as input to the random forest models in this study. Since the potential utility of the predictions of the models is for controlling a prosthetic ankle joint, the ankle angle and ankle moment were chosen as the output of our models. The performance of the inter-subject and subject-specific models were evaluated based on how well the trained models could generate the desired ankle angle and moment for thirty able-bodied subjects walking at five different speeds and two transtibial amputees (below-knee amputated) walking at self-selected comfortable and fast walking speeds. Additionally, a feature importance evaluation was performed using random forests to determine the most important features as input to the model in pursuit of reducing the input dimension while maintaining accuracy.

## 2. Methodology

Random forest models were trained to predict the ankle angle (θ_*ankle*_) and moment (τ_*ankle*_) using the ipsilateral hip flexion-extension angle (θ_*hip*_), knee flexion-extension angle (θ_*knee*_), shank segment orientation in the sagittal plane (θ_*shank*_), and their first derivatives $\left({\dot{\theta }}_{hip}\right),\left({\dot{\theta }}_{knee}\right),\left({\dot{\theta }}_{shank}\right)$. The training was performed using two different approaches: inter-subject and subject-specific. Normative input and output data were obtained from a publicly-available dataset (Fukuchi et al., 2018) while input data from two unilateral transtibial amputees was obtained experimentally. The experimental protocols were approved by the local ethics committee of the University Medical Center Göttingen, Göttingen, Germany (reference number: 26/3/18), and the participants gave their written informed consent before the experiment.

### 2.1. Subject Selection and Data Acquisition

Normative model inputs (θ_*hip*_, θ_*knee*_, and θ_*shank*_) and outputs (θ_*ankle*_ and τ_*ankle*_) were obtained from a publicly-available dataset (Fukuchi et al., 2018). The data was acquired using a marker-based motion capture system with 12 cameras (Raptor-4; Motion Analysis Corporation, Santa Rosa, CA, USA) and an instrumented treadmill (FIT; Bertec, Columbus, OH, USA). Twenty-two markers were placed on the pelvis, thigh, shank, and foot (Leardini et al., 2007). Out of this dataset, we selected 30 able-bodied volunteers' data (age: 39.7 ± 16.8 years, height: 167.5 ± 11.6 cm, mass: 67.1 ± 11.5 kg, 14 females) at five different walking speeds (1.04 ± 0.12, 1.22 ± 0.15, 1.4 ± 0.17, 1.58 ± 0.2, 1.77 ± 0.21 m/s) with a single trial at each speed. Each trial included a single gait cycle, which was defined by two consecutive heel contacts and time-normalized to 101 samples.

We also acquired data for two male unilateral transtibial amputees, both wearing energy-storage-and-return prostheses. Amputee 1 was 62 years old, 100 kg heavy, 1.85 m tall, amputated on the left, and wore a Pro-Flex prosthesis (Oessur, Iceland). Amputee 2 was 37 years old, 91 kg heavy, 1.75 m tall, amputated on the right, and wore a 1E95 Challenger prosthesis (Ottobock, Germany). Each amputee performed multiple trials of level ground walking at self-selected normal and fast speeds. Amputee 1 completed eight trials at both normal and fast walking speeds. Amputee 2 completed six trials at normal walking speed and four at fast walking speed. During each trial, three-dimensional motion data were recorded at 200 Hz using a motion capture system (Vicon Motion Systems, Ltd., UK) with retro-reflective markers, twelve infrared cameras, and data acquisition software (Nexus, Vicon Motion Systems, Ltd., UK). The markers were placed over the bony landmarks of the pelvis, thigh, shank, and foot. Compared to the marker set for the public dataset, we placed additional non-anatomical markers on the thigh and shank to ensure continuous tracking of these segments in a three-dimensional space. For each trial, data from one gait cycle, which was defined by two consecutive heel contacts on the amputated side, was considered for further analysis. The recorded marker trajectories were used to calculate θ_*hip*_, θ_*knee*_, and θ_*shank*_ on the amputated side. This was done using open-source biomechanical modeling software, OpenSim (Delp et al., 2007), and its generic musculoskeletal model (Gait 2392). Using the Scaling function of OpenSim and the marker trajectories from a separate static trial, the general model was modified to generate a unique model for each amputee. The unique models were used for the Inverse Kinematics and Body Kinematics Analysis functions of OpenSim to calculate θ_*hip*_, θ_*knee*_, and θ_*shank*_ within the gait cycle.

The data in the public dataset and the amputee data were both acquired in similar experiments using an infrared-based motion capture system, retro-reflective markers over bony landmarks of the lower extremities, and force plates to record ground reactions forces. For both the public dataset and our study, the kinematic and kinetic variables used as input features and model output were generated by performing inverse kinematics and dynamics on the motion capture data with a biomechanical model. Although the public dataset was generated for treadmill walking, it has been shown that treadmill gait is qualitatively and quantitatively similar to overground gait (Riley et al., 2007). Thus, we consider our experimental data and the public database chosen for our analyses to be comparable.

The able-bodied and amputee data were resampled to 200 samples per gait cycle, and their first derivatives, ${\dot{\theta }}_{hip},{\dot{\theta }}_{knee}$, and ${\dot{\theta }}_{shank}$ were computed as the numerical difference of angular positions between consecutive samples. Although the trials were resampled, the relative patterns of the kinematic and kinetic data during different speeds were preserved. The data were low-pass filtered using a Butterworth filter with a cut-off frequency of 6 Hz to remove noise (Little et al., 2013; Schurr et al., 2017; Mo et al., 2019).

### 2.2. Random Forest Model

Random forest is a supervised ensemble learning model that combines the predictions of multiple estimators called decision trees (Quinlan, 1986; Breiman, 2001), facilitating better overall performance. For regression, the prediction of a random forest model is the average of the predictions of all the decision trees in the ensemble.

In this study, a random forest regression model was trained to map the given θ_*hip*_, $\theta_{knee},\theta_{shank},{\dot{\theta }}_{hip},{\dot{\theta }}_{knee},$ and ${\dot{\theta }}_{shank}$ to the desired θ_*ankle*_ and τ_*ankle*_. The working principle of a random forest model can be summarized as follows:

1. Let *b* = 1..*B* be the number of random-forest estimators (trees) to be constructed.
2. For each random-forest tree, *T*_*b*_, a bootstrap sample, *S*, of size, *N*, is chosen from the training data. In this study, *N* equaled the number of data points in the training data.
3. *T*_*b*_ is constructed with *S*, by looping through the following steps for each node (the point at which a split takes place) of the tree until the maximum tree depth, *d*_*max*_, is reached:
  - *l* inputs are chosen from *k* input features in *S*, (*l* ≤ *k*). In this study *l* = *k* was chosen. This practice was justified empirically for regression problems in Geurts et al. (2006).
  - The best split among the *l* input features is chosen and the node is hence split into two child nodes. The best split is determined by an impurity function that measures the quality of a split. In this study, mean squared error was used as a criterion for measuring the quality of a split.
  To determine the best *d*_*max*_ and *B*, a grid search was performed on the training data with a 3-fold cross validation. The following parameter grid was used:
  (1) $$\begin{array}{r} \begin{array}{l} d_{max}\in \left\{4,6,8,10\right\}\\ B\in \left\{50,100,200,500\right\} \end{array} \end{array}$$
4. The ensemble of trees {*T*_1_..*T*_*B*_}, form the model. For regression, prediction, *f*(*r*′), at a new point *r*′ is the average of predictions of all the trees in the ensemble, i.e.,
  (2) $$\begin{array}{l} f\left(r^{\prime }\right)=1/B\overset{B}{\underset{b=1}{\sum }}T_{b}\left(r^{\prime }\right) \end{array}$$

The accuracy of predictions by the model was quantified using the coefficient of determination (*R*^2^) and root-mean-square error (*RMSE*) between the predicted output and the desired output.

### 2.3. Input Feature Combinations

Various combinations of input features were used for the two training approaches.

1. Inter-subject training:
  - Kinematic input features: $\theta_{hip},\theta_{knee},\theta_{shank},{\dot{\theta }}_{hip},{\dot{\theta }}_{knee},\text{and}{\dot{\theta }}_{shank}$
  - Kinematic + Anthropometric input features: $\theta_{hip},\theta_{knee},\theta_{shank},{\dot{\theta }}_{hip},{\dot{\theta }}_{knee},{\dot{\theta }}_{shank},$ height, mass, and age.
  - Important input features: Three most important input features for predicting θ_*ankle*_ and τ_*ankle*_.
2. Subject-specific training:
  1. Kinematic input features : $\theta_{hip},\theta_{knee},\theta_{shank},{\dot{\theta }}_{hip},{\dot{\theta }}_{knee},\text{and}{\dot{\theta }}_{shank}$
  2. Important input features: Three most important input features for predicting θ_*ankle*_ and τ_*ankle*_.

The importance of a feature, *l*′, in *T*_*b*_ is calculated using the mean decrease of impurity (MDI) (Breiman et al., 1984) as:

(3) $$\begin{array}{l} Imp\left(T_{b},l^{\prime }\right)=\frac{\sum_{j\in M\left(l^{\prime }\right)}MDI}{\sum_{j\in M}MDI} \end{array}$$

where *M* is the set of all nodes in a tree and *M*(*l*′) represents the set of all nodes in a tree that are split based on *l*′. The relative importance of input features was obtained by averaging the feature importance for all the trees in the ensemble.

(4) $$\begin{array}{l} Imp\left(l^{\prime }\right)=\frac{1}{B}\overset{B}{\underset{b=1}{\sum }}Imp\left(T_{b},l^{\prime }\right) \end{array}$$

### 2.4. Inter-subject Model

**Figure 1:**
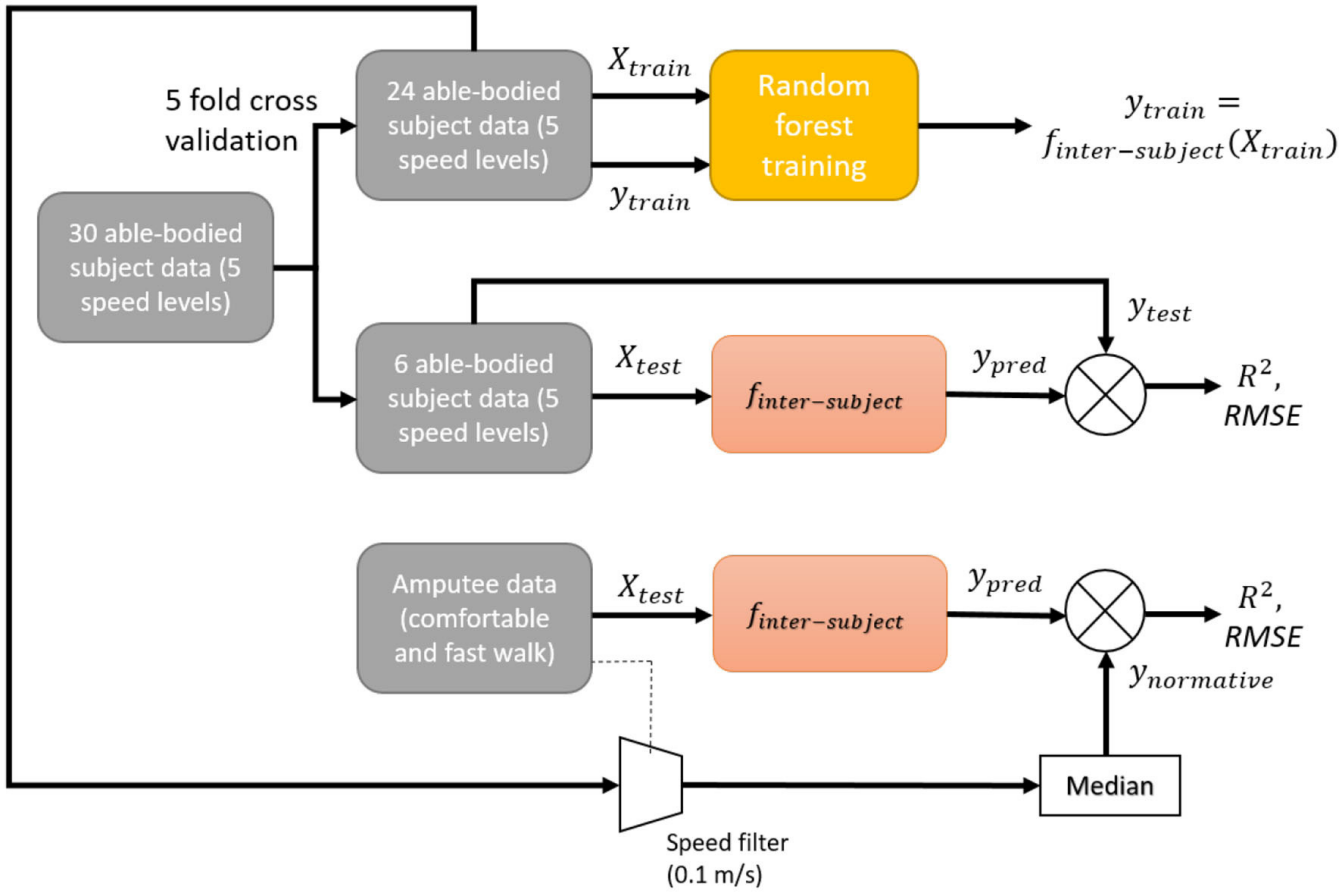
Block diagram of training and validation procedure of an inter-subject model on able-bodied and amputee subjects.

The training and validation procedures of an inter-subject model on able-bodied and amputee subjects are shown in Figure 1. The training data was obtained from a publicly-available dataset (Fukuchi et al., 2018). The trained models were validated using 5-fold cross-validation: the inter-subject model was trained with data from 24 out of the 30 able-bodied subjects, with the remaining six subjects used for validation. For each subject in the training data, trials at five different speeds were used to generalize the model. For validation, we compared the inverse kinematics and inverse dynamics derived θ_*ankle*_ and τ_*ankle*_ against their predicted values.

The trained model was also tested by assessing its predictions based on inputs from the two transtibial amputees. The predictions from amputee inputs were compared against the corresponding normative outputs, which were the median of outputs from able-bodied subjects whose walking speeds were within 0.1m/s of that of the amputee's mean walking speed. The normative outputs were generated from 24 out of the 30 able-bodied subjects in the same manner as the aforementioned 5-fold cross-validation. We have chosen the median for computing the normative values as it is less affected by outlier gait patterns than the mean value. The walking speed was estimated from the speed of the pelvis in the walking direction. The test output was obtained from the left and right legs of able-bodied subjects for Amputees 1 and 2, respectively.

The training of an inter-subject model can be formalized as learning a function *f*_*inter*−*subject*_ such that:

(5) $$\begin{array}{l} \overset{S}{\underset{s=1}{⋃}}\overset{T}{\underset{t=1}{⋃}}{\text{y}}_{able-bodied}^{s,t}=f_{inter-subject}\left(\overset{S}{\underset{s=1}{⋃}}\overset{T}{\underset{t=1}{⋃}}{\text{X}}_{able-bodied}^{s,t}\right) \end{array}$$

where ${\text{y}}_{able-bodied}^{s,t}\in {\mathbb{R}}^{N\times 2}$ are the predicted θ_*ankle*_ and τ_*ankle*_ in a gait cycle (containing *N* observations) of *t*-th trial of the *s*-th able-bodied subject, ${\text{X}}_{able-bodied}^{s,t}\in {\mathbb{R}}^{N\times m}$ are the input features of dimension *m* within the gait cycle (containing *N* observations) of the *t*-th trial of the *s*-th able-bodied subject and *f*_*inter*−*subject*_ is a random forest based inter-subject model.

The error in predictions of an inter-subject model for the *k*-th trial of a test subject is given by:

(6) $$\begin{array}{l} \varepsilon_{subject}^{k}=f_{inter-subject}\left({\text{X}}_{subject}^{k}\right)-{\text{y}}_{subject}^{k} \end{array}$$

where ${\text{X}}_{subject}^{k}\in {\mathbb{R}}^{N\times m}$ are the input features of dimension *m* and ${\text{y}}_{subject}^{k}\in {\mathbb{R}}^{N\times 2}$ are the desired θ_*ankle*_ and τ_*ankle*_ within the gait cycle (containing *N* observations). For an amputee, the normative value for his *k*-th trial can be calculated as

(7) $$\begin{array}{l} {\text{y}}_{subject}^{k}={\text{y}}_{normative}^{k}=\text{median}\left({\text{y}}_{able-bodied}^{s,j_{k}}\right),s=1..S, \end{array}$$

where *j*_*k*_ is the index of the trial of an able-bodied subject, *s*, where the mean pelvis velocity of the subject was within ±0.1*m*/*s* of that of amputee's *k*-th trial.

### 2.5. Subject-Specific Model

**Figure 2:**
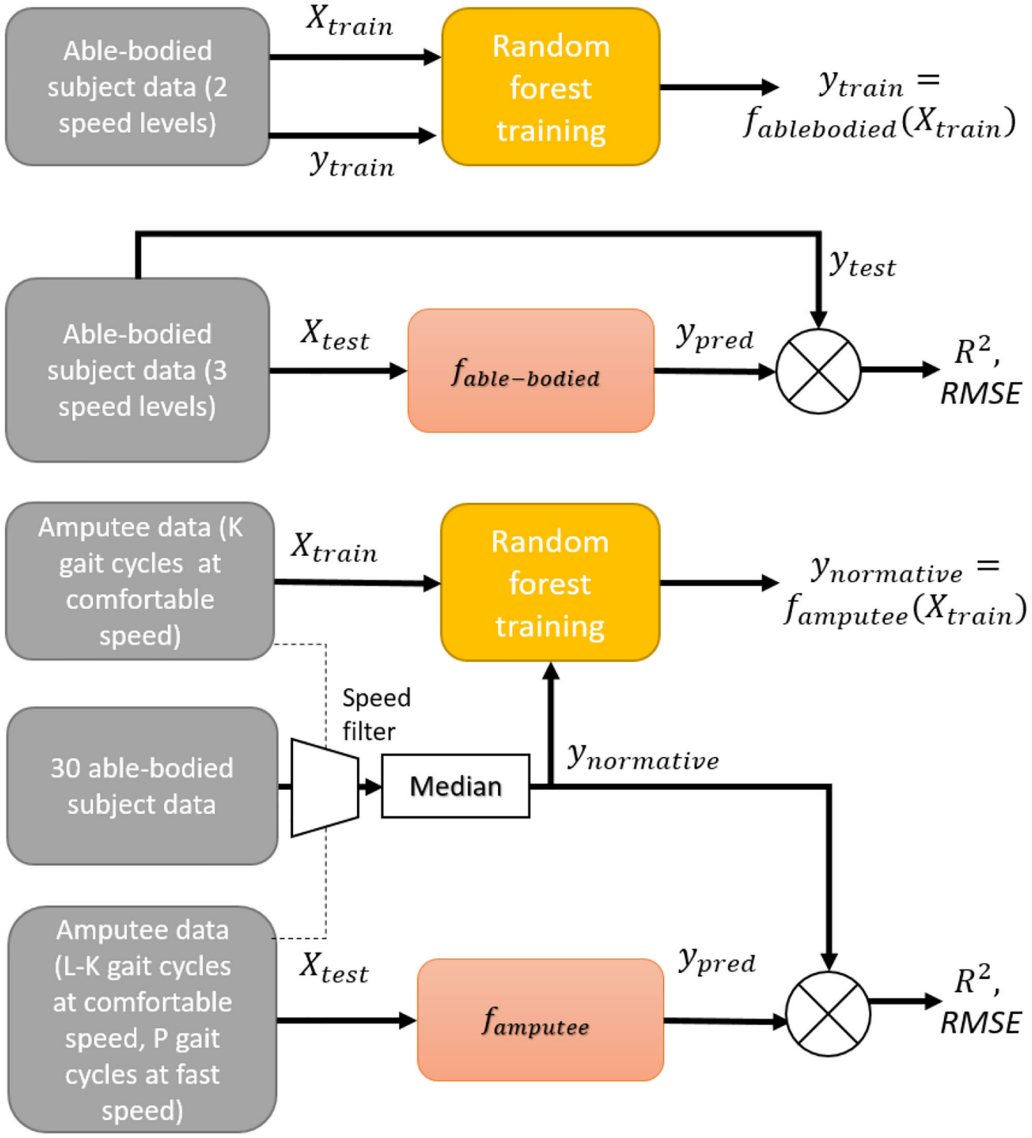
Block diagram of training and validation procedures of subject-specific models for able-bodied and amputee subjects. For Amputee 1, L = 8, K = 5, and P = 8. For Amputee 2, L = 6, K = 4, and P = 4.

The training and validation procedures of a subject-specific model are illustrated in Figure 2. A subject-specific model was trained separately for each of the 30 able-bodied subjects. For each subject, the model was trained using data from trials at the second and fourth speed levels and tested with data from trials at the remaining three speeds. Since our goal was to predict the ankle angle and moment over a wide range of walking speeds using as little training data as possible, we chose the speeds at a level lower and higher than self-selected comfortable speeds. By doing so, we attempted to train the model with trajectories from a minimal number of speeds which would best describe all the speeds in the dataset. The training of a subject-specific model can be formulated as learning a function, *f*_*subject*_,

(8) $$\begin{array}{l} \overset{T}{\underset{t=1}{⋃}}{\text{y}}_{subject}^{t}=f_{subject}\left(\overset{T}{\underset{t=1}{⋃}}{\text{X}}_{subject}^{t}\right) \end{array}$$

where ${\text{X}}_{subject}^{t}\in {\mathbb{R}}^{N\times m}$ and ${\text{y}}_{subject}^{t}\in {\mathbb{R}}^{N\times 2}$. The prediction error for the *k*-th trial is given by

(9) $$\begin{array}{l} \varepsilon_{subject}^{k}=f_{subject}\left({\text{X}}_{subject}^{k}\right)-{\text{y}}_{subject}^{k} \end{array}$$

For transtibial amputees, a model was trained to learn a relationship between amputee's input features during normal speed walking and the normative θ_*ankle*_ and τ_*moment*_ of able-bodied individuals. The relationship learned by a subject-specific model trained for a transtibial amputee is given by $f_{amputee}:{\mathbb{R}}^{M\times m}\to {\mathbb{R}}^{M\times 2}$ such that,

(10) $$\begin{array}{l} \overset{T}{\underset{t=1}{⋃}}{\text{y}}_{normative}^{t}=f_{amputee}\left(\overset{T}{\underset{t=1}{⋃}}{\text{X}}_{amputee}^{t}\right) \end{array}$$

where ${\text{y}}_{normative}^{t}$ is computed from able-bodied subjects, whose speed was within 0.1 m/s of that of the amputee.

The trained model was then validated using a leave-K-out cross-validation strategy, in which *K* gait cycles of normal speed walking data were used for training and remaining *L* − *K* gait cycles of normal and *P* gait cycles of fast speed walking data were used for validation. For Amputee 1, *L* = 8, *K* = 5, and *P* = 8. For Amputee 2, *L* = 6, *K* = 4, and *P* = 4. The error in prediction for *k*-th trial of an amputee is given by.

(11) $$\begin{array}{l} \varepsilon_{amputee}^{k}=f_{amputee}\left({\text{X}}_{amputee}^{k}\right)-{\text{y}}_{normative}^{k} \end{array}$$

### 2.6. Statistical Analyses

In order to compare the two training approaches as well as the outcomes of the different feature combinations, we performed statistical significance tests. To analyze the significant difference between feature combinations, a Wilcoxon signed-rank test was performed between each pair of feature combinations for both the inter-subject and the subject-specific approaches averaged across all speed levels for both able-bodied and amputee subjects. To analyze the significant difference between the two training approaches, we performed another Wilcoxon signed-rank test to compare the accuracies for each feature combination for able-bodied and amputee subjects averaged across all trials speed levels. The significance level for both the tests was 0.05.

## 3. Results

### 3.1. Input Features

**Figure 3:**
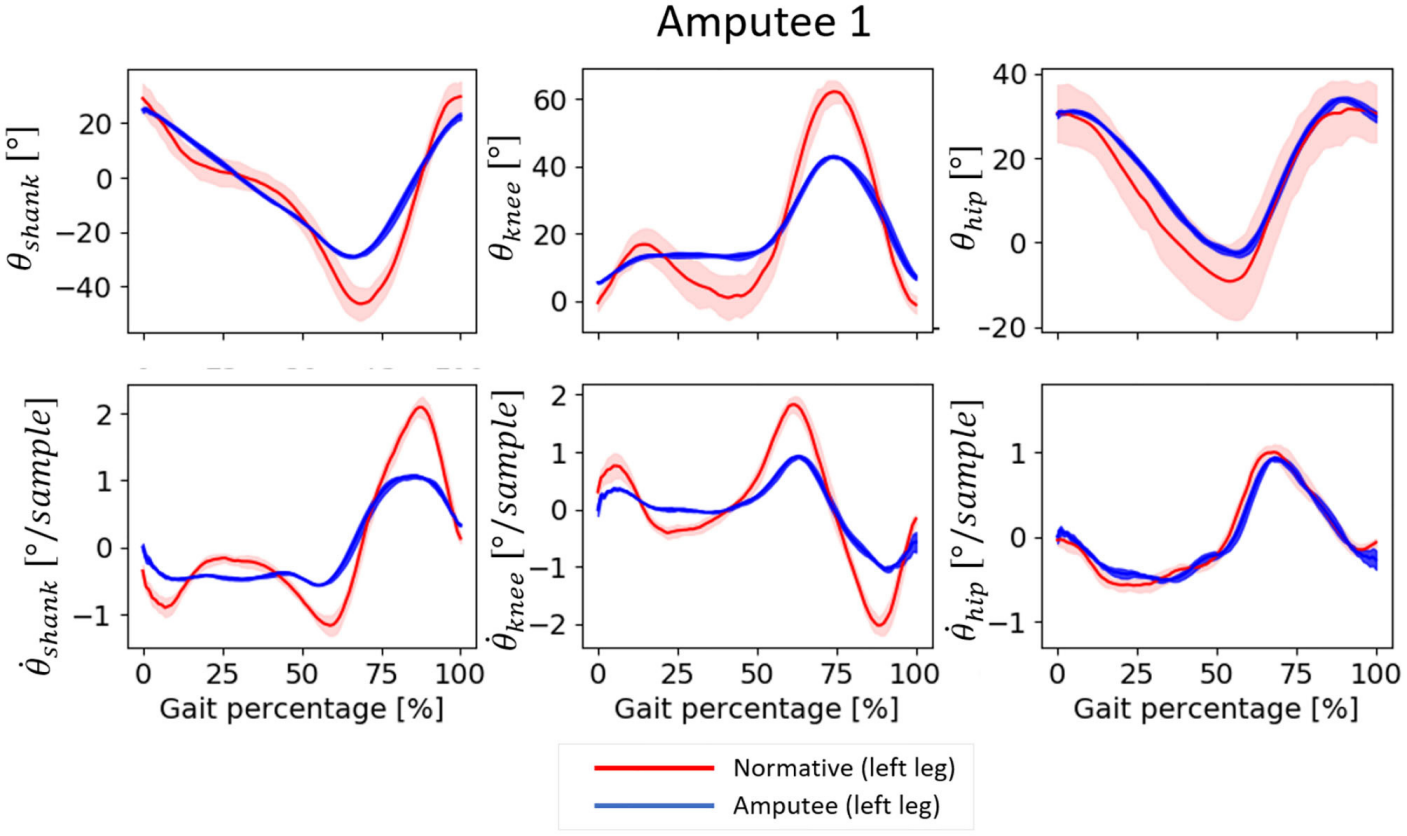
Input patterns for one gait cycle (measured between two consecutive heel contact of the same leg) from Amputee 1 and the corresponding normative data for normal speed walking. 0–60% represents the stance phase and 60–100% represent the swing phase of the gait. The normative values are computed from the data of able-bodied subjects walking at a speed within 0.1 m/s as that of Amputee 1. Positive θ_*shank*_ indicates forward rotation, positive θ_*knee*_ indicates knee flexion, and positive θ_*hip*_ indicates hip flexion, in the direction of motion, i.e., in the sagittal plane. The shaded regions are the median absolute deviation from the median.

**Figure 4:**
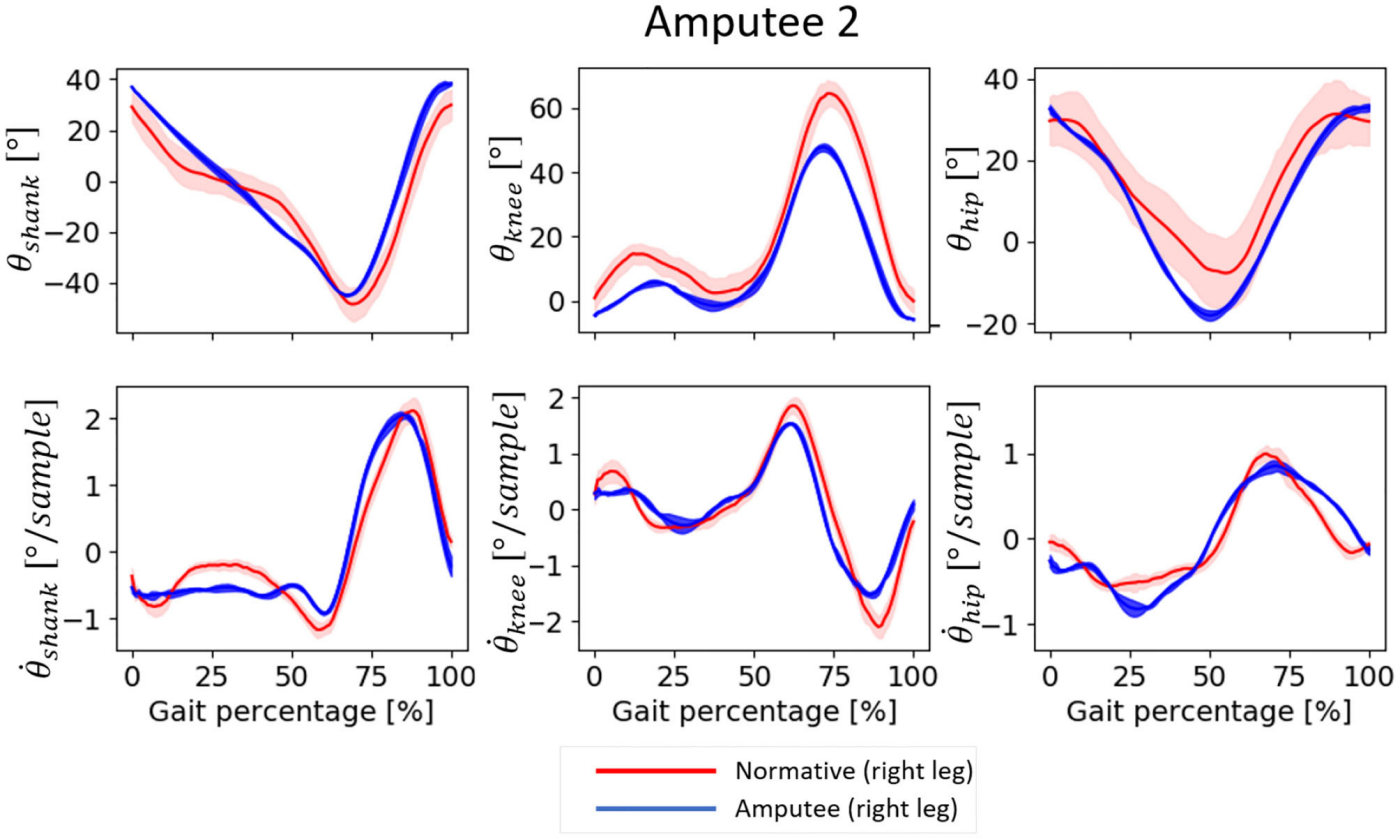
Input patterns for one gait cycle (measured between two consecutive heel contact of the same leg) from Amputee 2 and the corresponding normative data for normal speed walking. 0–60% represents the stance phase and 60–100% represent the swing phase of the gait. The normative values are computed from the data of able-bodied subjects walking at a speed within 0.1 m/s as that of Amputee 2. Positive θ_*shank*_ indicates forward rotation, positive θ_*knee*_ indicates knee flexion, and positive θ_*hip*_ indicates hip flexion, in the direction of motion, i.e., in the sagittal plane. The shaded regions are the median absolute deviation from the median.

Both amputees showed deviations in the temporal patterns of the input features measured from their amputated sides during normal walking trials. Amputee 1 showed reduced forward rotation of the shank and reduced knee flexion, mainly at the end of the stance phase and the beginning of the swing phase, compared to normative data (Figure 3). The corresponding first derivatives, ${\dot{\theta }}_{shank}$ and ${\dot{\theta }}_{knee}$, showed reduced range compared to the normative data. Conversely, the trajectories of the θ_*hip*_ and its derivative aligned more closely with the corresponding normative values. Amputee 2 also showed deviations from normative data, but the magnitude of deviation appeared to be smaller compared to Amputee 1 (Figure 4). Amputee 2 showed reduced knee flexion and increased hip extension compared to the normative data.

### 3.2. Inter Subject Model

#### 3.2.1. Able-Bodied Test Subjects

**Figure 5:**
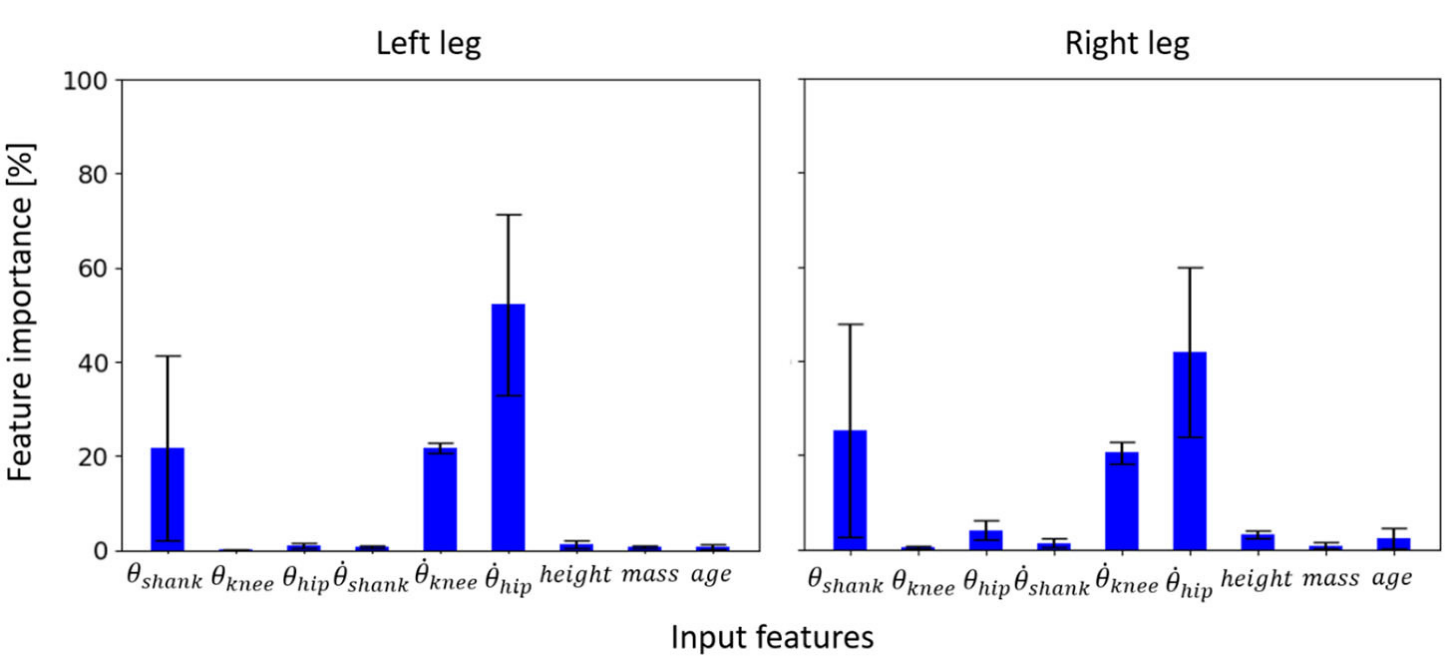
Feature importance for the inter-subject model to learn the input-output relationship for the left and right legs of able-bodied subjects walking at different speeds. Error bars are the standard deviations obtained from cross-validation.

It was observed that for the inter-subject model trained to learn the input-output relationship using data from 24 able-bodied subjects, three input features (out of six kinematic and three anthropometric inputs), θ_*shank*_, ${\dot{\theta }}_{knee}$, and ${\dot{\theta }}_{hip}$, were found to be the most informative on both left and right sides (Figure 5). The anthropometric inputs had little relative importance in the model's prediction of θ_*ankle*_ and τ_*ankle*_.

**Figure 6:**
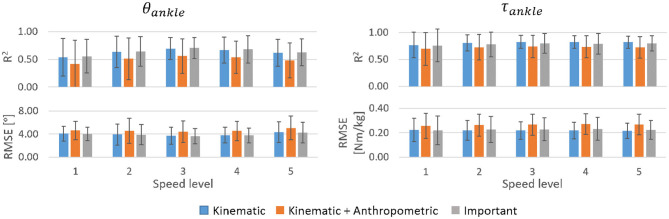
*R*^2^ and *RMSE* for θ_*ankle*_ and τ_*ankle*_ predictions by the inter-subject model for different walking speeds averaged across able-bodied test subjects. Error bars are the inter-individual standard deviation.

In most cases, the six kinematic features and the three most important features achieved similar prediction accuracies for both θ_*ankle*_ and τ_*ankle*_ while the addition of anthropometric features impaired prediction (Figure 6). The τ_*ankle*_ predictions generally showed higher accuracy than θ_*ankle*_ predictions. For θ_*ankle*_ predictions, the mean *R*^2^ peaked at the medium speed (mean *R*^2^ = 0.70 using the three important input features). A similar trend was also observed for τ_*ankle*_ prediction though not to the same extent (mean *R*^2^ at medium speed = 0.80 using the three important input features). Generally, the *RMSE* changed inversely to the corresponding *R*^2^.

#### 3.2.2. Amputee Test Subjects

The inter-subject models were used to predict the θ_*ankle*_ and τ_*ankle*_ for the two transtibial amputees during normal and fast walking speeds and predictions were compared against normative values calculated from able-bodied subjects' data at walking speeds within ±0.1m/s as that of the amputee subject. The speed of Amputee 1 was 1.34 ± 0.03 and 1.56 ± 0.02 m/s for normal and fast walking, respectively. The corresponding values for Amputee 1's able-bodied counterparts were 1.32 ± 0.05 and 1.55 ± 0.05 m/s. The speed of Amputee 2 was 1.2 ± 0.01 and 1.61 ± 0.05 m/s, and the corresponding values for Amputee 2's able-bodied counterparts were 1.22 ± 0.05 and 1.62 ± 0.05 m/s.

**Figure 7:**
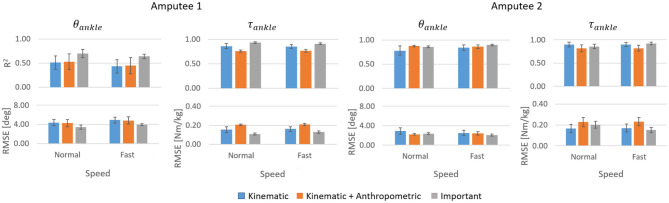
Performance of inter-subject model for normal and fast walking datasets of Amputees 1 and 2. Error bars are the inter-trial standard deviation.

**Figure 8:**
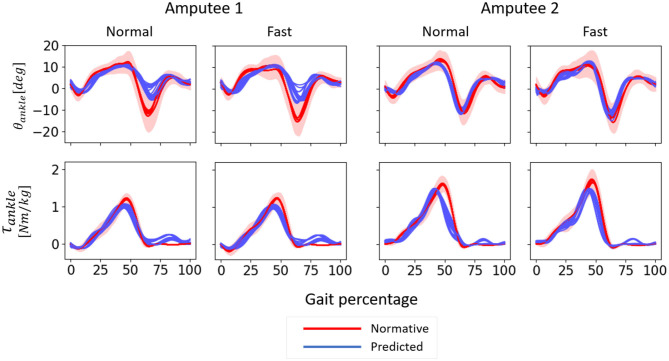
Inter-subject model predicted values of θ_*ankle*_ and τ_*ankle*_ for each gait cycle of normal-speed and fast-speed walking for all the cross-validation iterations for Amputees 1 and 2 using only the three most important input features. One gait cycle was defined as the time between two consecutive heel contacts of the amputated leg. 0–60% of gait percentage indicates stance phase and 60–100% of the gait percentage indicates swing phase. Positive angle indicates dorsiflexion, and positive moment indicates plantarflexion. The normative θ_*ankle*_ and τ_*ankle*_ were calculated from able-bodied data used for training the inter-subject model. The shaded area indicates one median absolute deviation from the median.

For both amputees, the prediction accuracies (Figure 7) were slightly higher compared to that of the able-bodied individuals. For Amputee 1, τ_*ankle*_ predictions (*R*^2^ = 0.93 for normal speed walking and 0.91 for fast speed walking) were more accurate compared to θ_*ankle*_ (*R*^2^ = 0.70 for normal speed walking and 0.64 for fast speed walking) with the three most important input features. Regardless of speed, using only the three most important input features resulted in more accurate predictions for both θ_*ankle*_ and τ_*ankle*_ compared to using all six kinematic features. Similar to the predictions for able-bodied individuals (Figure 6), inclusion of the anthropometric inputs generally impaired prediction accuracy. For Amputee 2, both θ_*ankle*_ and τ_*ankle*_ gave high accuracies for both normal and fast speed walking (for normal speed, *R*^2^ = 0.86 for both θ_*ankle*_ and τ_*ankle*_ and for fast walking were *R*^2^ = 0.90 for θ_*ankle*_ and 0.92 for τ_*ankle*_). The trajectories of the θ_*ankle*_ and τ_*ankle*_ predicted by the inter-subject model for the amputees deviated from the normative trajectories at certain sections of the gait cycle (Figure 8). For Amputee 1, the predicted trajectory of θ_*ankle*_ deviated substantially from the desired normative trajectory during late stance and early swing at both speeds. The deviations resulted in reduced peak plantarflexion during push-off. These kinematic deviations were accompanied by slightly reduced peak push-off plantarflexor moment and increased plantarflexor moment during swing. For Amputee 2, predicted θ_*ankle*_ closely matched the normative trajectory compared to Amputee 1 for both speeds. This was also evident from the high *R*^2^ score of θ_*ankle*_ prediction for Amputee 2 than for Amputee 1 (Figure 7). The τ_*ankle*_ predictions for Amputee 1 showed deviations from the normative values during the mid-stance as well as during the mid-swing phase for both speeds. The τ_*ankle*_ predictions for Amputee 2 also showed similar deviations from normative values but of slightly higher amplitude than Amputee 1 during normal speed.

### 3.3. Subject Specific Models

#### 3.3.1. Able-Bodied Subjects

**Figure 9:**
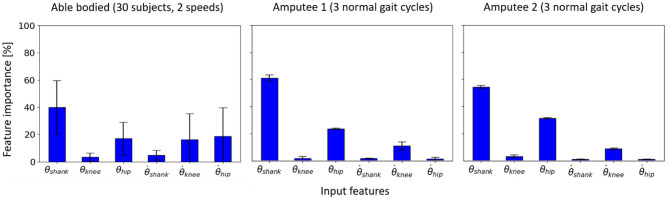
Feature importance for the subject-specific models trained using data from two (out of five) speed trials of 30 able-bodied subjects and three normal speed gait cycles of Amputee 1 and 2 (i.e., 6- and 4-fold cross-validation, respectively).

The random forest models trained individually for 30 able-bodied subjects using subject-specific approach recorded θ_*shank*_ as the most important feature on average across all subjects, followed by θ_*hip*_ and ${\dot{\theta }}_{hip}$ (Figure 9). ${\dot{\theta }}_{knee}$ also had an average importance comparable to ${\dot{\theta }}_{hip}$. The importance of ${\dot{\theta }}_{hip}$, which was the highest for the inter-subject model (Figure 5), was substantially reduced.

**Figure 10:**
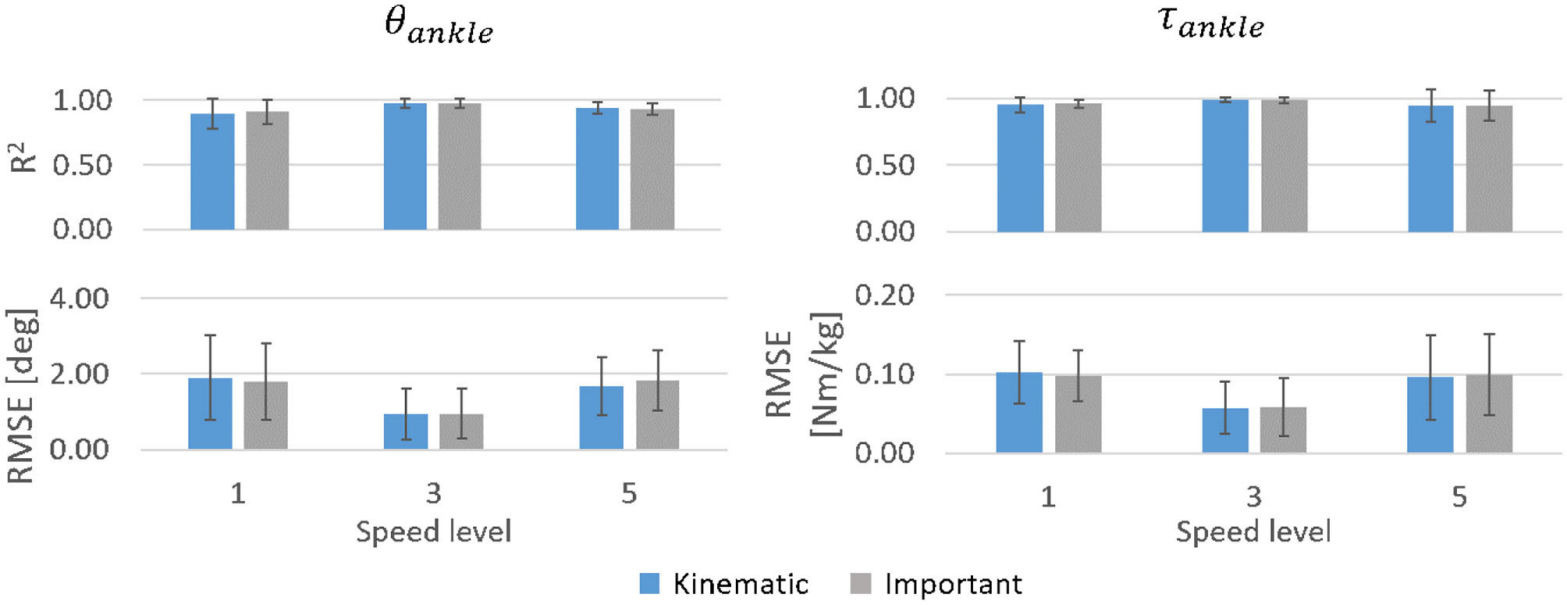
*R*^2^ and *RMSE* for θ_*ankle*_ and τ_*ankle*_ predicted by the subject-specific models for three tested speed levels averaged across 30 able-bodied subjects. Error bars show inter-subject standard deviation.

The subject-specific models substantially outperformed the inter-subject model in predicting θ_*ankle*_ and τ_*ankle*_ regardless of the speed level (Figure 10). Mean *R*^2^ exceeded 0.90 for θ_*ankle*_ and 0.94 for τ_*ankle*_ across the tested speeds. The mean *RMSE* was below 2° and 0.11 Nm/kg for the θ_*ankle*_ and τ_*ankle*_, respectively, across the tested speeds. Similar to the inter-subject model trained for different speed levels, the highest accuracy was obtained in the mid-speed level. Both the kinematic and important input features gave similar accuracy of predictions.

#### 3.3.2. Amputee Subjects

For both the transtibial amputees, the three most important input features were θ_*shank*_, θ_*hip*_, and ${\dot{\theta }}_{knee}$ which were also found to be important for subject-specific models trained for able-bodied subjects (Figure 9). However, ${\dot{\theta }}_{hip}$ which was found to be important in able-bodied subjects were not important for the transtibial amputees.

**Figure 11:**
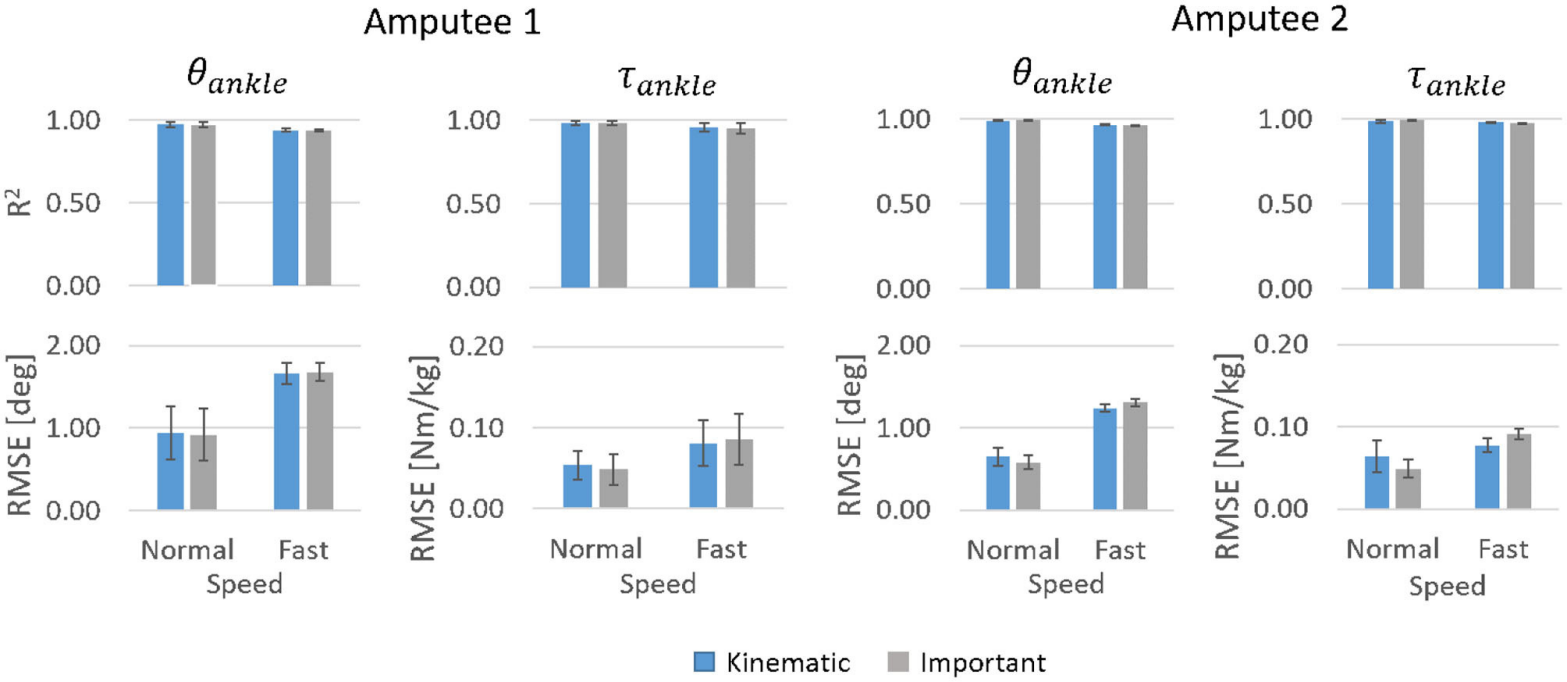
*R*^2^ values and *RMSE* for θ_*ankle*_ and τ_*ankle*_ predictions by subject-specific models for normal and fast walking datasets of Amputees 1 and 2. Error bars show inter-trial standard deviation.

The subject-specific models trained for amputees gave very high accuracy for both θ_*ankle*_ and τ_*ankle*_ predictions. For both Kinematic and Important input features, mean *R*^2^ for θ_*ankle*_ for Amputee 1 was above 0.97 (mean *RMSE* ≤ 1°) for normal walking and above 0.94 (mean *RMSE* ≤ 1.7°) for fast walking. Mean *R*^2^ for τ_*ankle*_ was above 0.98 (mean *RMSE* ≤ 0.05 Nm/kg) for normal walking and above 0.95 (mean *RMSE* ≤ 0.09 Nm/kg) for fast walking (Figure 11). For Amputee 2, mean *R*^2^ for θ_*ankle*_ was above 0.98 (mean *RMSE* ≤ 0.7°) for normal walking and above 0.95 (mean *RMSE* ≤ 1.3°) for fast walking. Mean *R*^2^ for τ_*ankle*_ was above 0.98 (mean *RMSE* ≤ 0.06 Nm/kg) for normal walking and above 0.97 (mean *RMSE* ≤ 0.09 Nm/kg) for fast walking.

**Figure 12:**
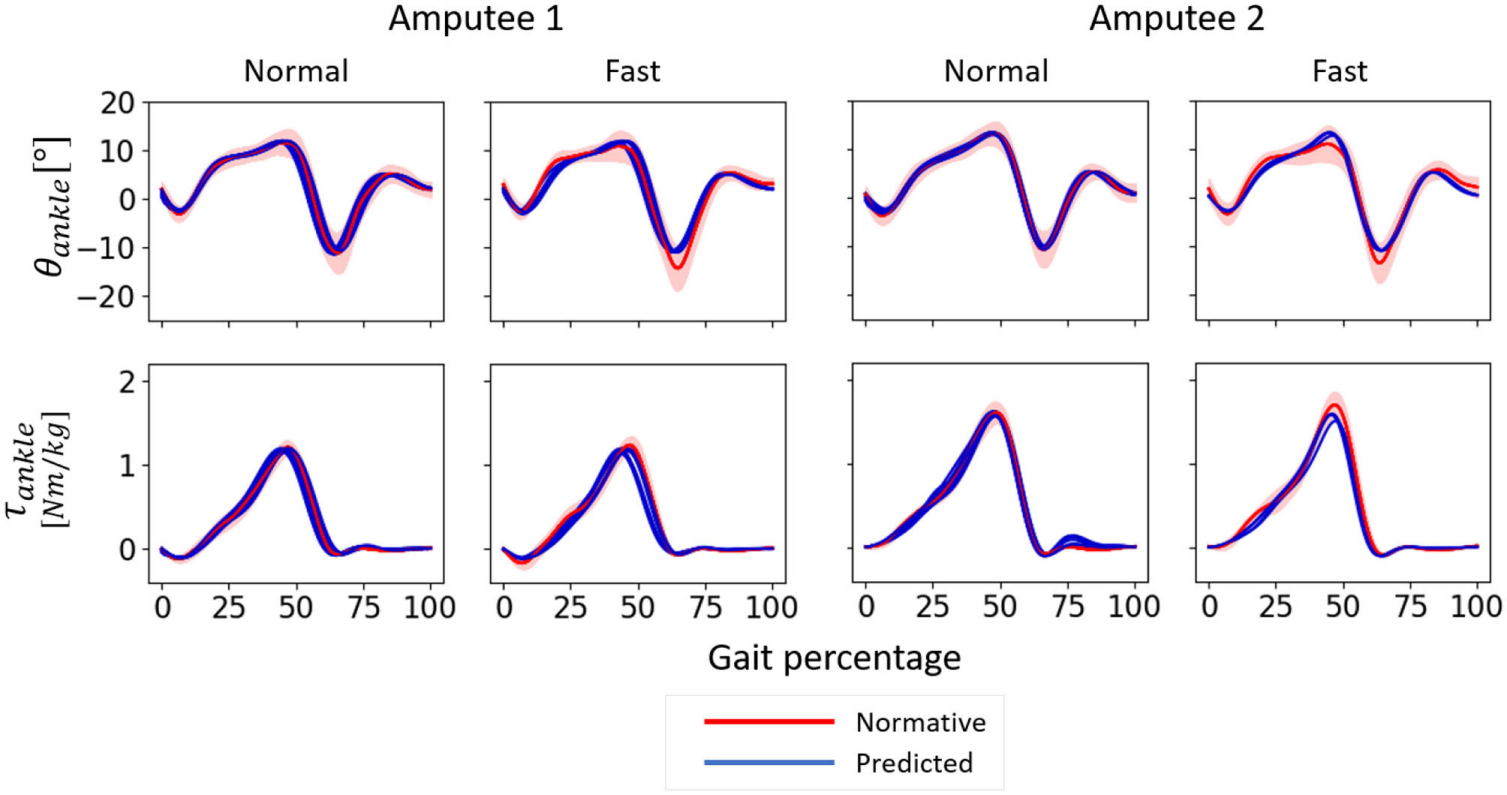
θ_*ankle*_ and τ_*ankle*_ predicted by subject-specific models for normal-speed and fast-speed walking datasets of Amputees 1 and 2 using only the three most important input features. One gait cycle was defined as the time between two consecutive heel contacts of the amputated leg. 0–60% of the gait percentage represents the stance phase and 60–100% of the gait percentage represent the swing phase. Positive angle indicates dorsiflexion, and positive moment indicates plantarflexion. The models were trained using three gait cycles of normal speed walking. The normative values were calculated from able-bodied subjects using trials at speeds within 0.1 m/s as that of each amputee. The shaded area indicates one median absolute deviation from the median.

At a comfortable speed, subject-specific models performed slightly better with only the three most important input features compared to using all six kinematic features whereas this trend was reversed at fast speed. Regardless of the input features, the predictions of θ_*ankle*_ and τ_*ankle*_ were more accurate at a comfortable speed for both amputees. The temporal patterns of θ_*ankle*_ and τ_*ankle*_ predicted by the subject-specific models followed normative patterns very closely albeit a slight deviation of θ_*ankle*_ prediction during terminal stance and initial swing phase of fast walking (Figure 12).

### 3.4. Statistical Comparisons

**Figure 13:**
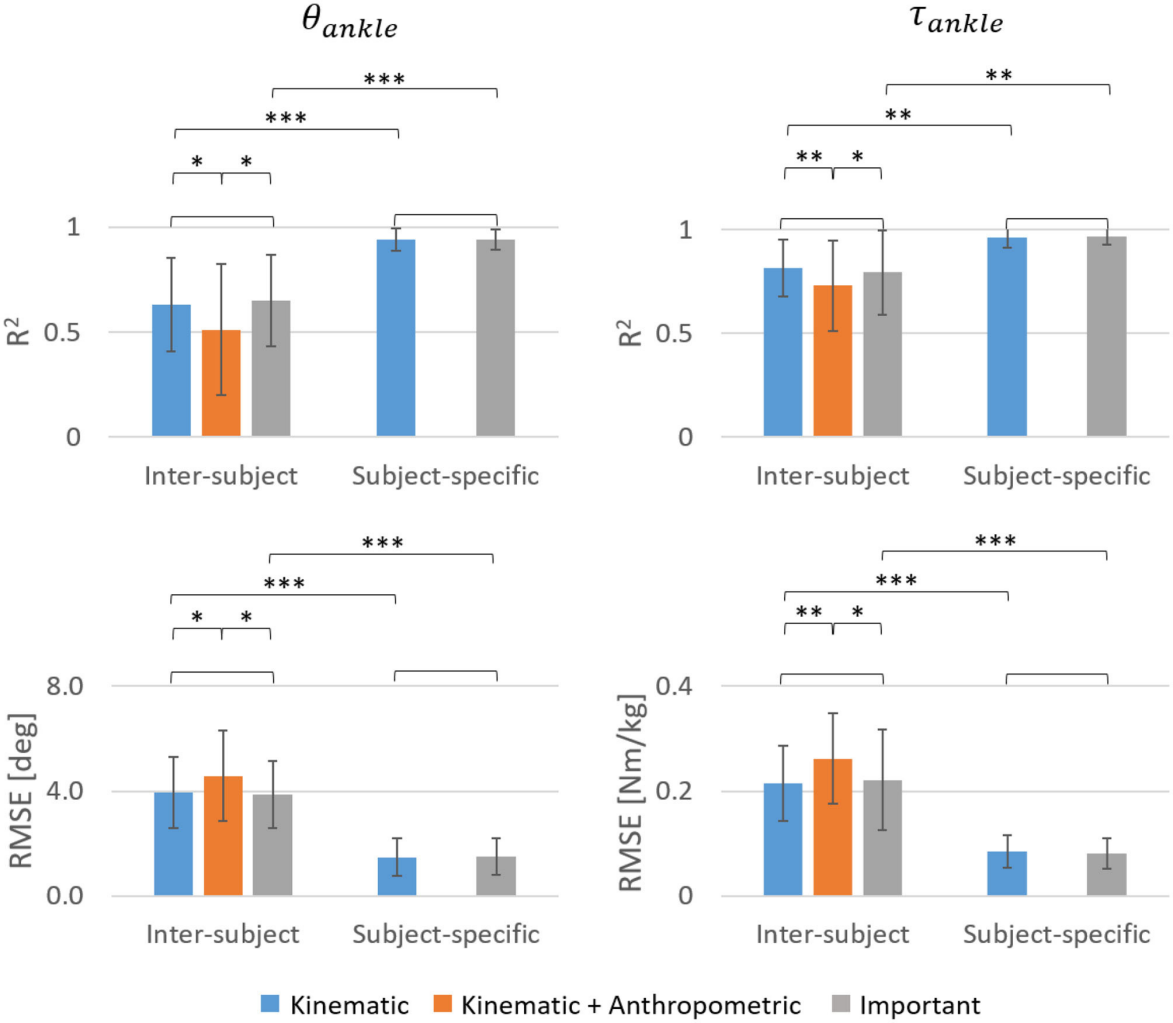
Statistical comparison between the *R*^2^ and *RMSE* values averaged across trails and speed levels of two amputees and 30 able-bodied subjects. A Wilcoxon signed-rank test was performed between each of the feature combinations for both inter-subject and subject-specific approaches and also between the inter-subject and subject-specific approaches for kinematic and important input features (no star: *p* > 0.05, ^*^p < 0.01, ^**^p < 1*e*^−5^, ^***^p < 1*e*^−6^).

A statistical comparison between the different input feature combinations showed that for the inter-subject model, the *R*^2^ of θ_*ankle*_ and τ_*ankle*_ were significantly higher and corresponding *RMSE* values significantly lower while using either the Kinematic or Important input features compared to using the Kinematic + Anthropometric input features (Figure 13). There was no significant difference in accuracy between the Kinematic and Important input features for both inter-subject and subject-specific models. Statistical comparison between the inter-subject and subject-specific training approaches showed that the subject-specific model performed significantly better in predicting both θ_*ankle*_ and τ_*ankle*_ using either the Kinematic or Important input features.

## 4. Discussion

In this study, we examined how the type of training affects the performance of a random forest model when predicting θ_*ankle*_ and τ_*ankle*_ for 30 able-bodied and two unilateral transtibial amputees from the residual limb kinematics during walking. The random forest models were trained using two different approaches: inter-subject and subject-specific. The inter-subject approach seeks a generalized input-output relationship for a group of individuals while the subject-specific approach establishes a unique input-output relationship for each individual. Since a purely subject-specific model is not possible for the transtibial amputees due to unavailability of the desired reference output (ankle angle and ankle moment), we proposed a modified subject-specific model to map the residual limb kinematics of the amputees to a desired normative trajectory of ankle angle and moment derived from a group of able-bodied individuals.

As expected, the inter-subject model exhibited several flaws in its performance. The inaccuracy of the inter-subject model may have been due to the deviation of amputee input features from the able-bodied training data. Amputee 1 showed substantial deviations in two of the three most important input features: θ_*shank*_ and ${\dot{\theta }}_{knee}$ (Figure 3), while Amputee 2 showed less deviation for the three most important input features (Figure 4), and this may have resulted in the relatively inaccurate prediction of θ_*ankle*_ for Amputee 1 (Figure 8). Also, it was observed that the inclusion of the anthropometric inputs generally impaired prediction accuracy, most probably due to inconsistent relationships between the anthropometric inputs and the predicted variables. This may have been due to our dataset structure which contained only one trial per walking speed for each subject. Therefore, the effect of anthropometric inputs should be further investigated on a dataset that contains more trials of the same speed levels or in a dataset where inter-speed variation for each subject is lower.

The subject-specific models performed significantly better than the inter-subject model for both able-bodied individuals and amputees. For both the able-bodied individuals and the two amputees, high prediction accuracy was obtained for both θ_*ankle*_ and τ_*ankle*_ for all the tested speed levels (Figures 10, 11). Using the kinematic input features or the important input features gave comparable performance. This finding indicates that subject-specific models may be more ideal for devising a potential control strategy for an intelligent prosthesis than the inter-subject models.

One of the most important results from our study is the ability of the subject-specific model to generalize its learning to different untrained walking speeds with high accuracy. For a real-world implementation of a control scheme using the proposed model, this is a very important characteristic. We tested the speed generalization capability of the subject-specific model for three untrained speeds for able-bodied subjects and one untrained speed for each of the amputee subjects. The results suggest that if the model is trained for a certain speed, it can generalize this learning up to a speed difference of 0.4 m/s.

It was also shown in our study that, using only the important features selected by the random forest as input, it was possible to obtain similar or higher accuracy of the predicted output variables compared to using all the available input features. Here, we also point out that our models did not have any physiological basis. Therefore, the important features are not necessarily the most important determinants of gait. Instead, the important input features are those variables that most effectively discerned the outputs for model learning. Specifically, the important input features are used preferentially by random forests to split at a given node and result in larger variance reduction of the output values on the left and the right nodes compared to other features. It can be interpreted that, important features define an input-output relation with minimal overlap in input values for different values of the output. The features marked as the most important were different for inter-subject and subject-specific models, which we assume is due to the difference in the data variations in training approaches. However, θ_*shank*_ remained important for both approaches. The large standard deviations of feature importance for subject-specific models trained for able-bodied subjects indicated large inter-individual differences in relative feature importance. This could be due to the inter-individual variations in gait patterns of able-bodied subjects. More interestingly, the features which were determined as important were consistent between the two amputee subjects. Although the input features of the amputees deviate from each other, this similarity may be partly due to the training approach we followed to map the amputee's input features to normative outputs. A limitation of the feature importance calculation using random forest's mean decrease in impurity is that the correlated features are given similar but reduced relative importance. However, this did not affect the prediction performance of our models while using Important input features, since the importance of these features summed to at least 85%. Therefore, our results suggest that feature selection could result in high accuracy while reducing the input dimension thereby potentially increasing model efficiency for real-time applications.

We have chosen a very heterogeneous dataset with respect to subjects, trials, and speeds for inferring the able-bodied model inputs and outputs. This was done to simulate a real-world scenario to some extent, where the gait cycles across trials and speeds might not be highly homogeneous. We may consider the heterogeneity across subjects and speeds as simulating variations that require the model to be robust. Thus, we have tested most of our training and validation conditions in adverse and minimal data conditions where we had only one trial per speed for each of five speeds for thirty able-bodied subjects. We believe that much better performance of the models could be achieved if a more homogeneous dataset or a dataset with more trials from the same speed level would be considered. Also, our models were based on pattern recognition. Therefore, the actual or the physical meaning of the predictor input variables were not necessary as long as the relative patterns were preserved. For example, the first derivatives were calculated as the numerical difference between the consecutive angular positions without time normalization.

The performance of the subject specific training approach was comparable to other state-of-the-art machine learning regression algorithms used in previous studies for gait variable predictions. For example, Eslamy and Schilling (2018) reported an *R*^2^ score above 0.92 using Gaussian process regression for ankle kinematic prediction for the trained speed levels. Ardestani et al. (2014) used a wavelet neural network for prediction for lower extremity moments during walking and obtained a correlation coefficient, ρ > 0.94, and *NRMSE* < 10%. Dey et al. (2019) reported *R*^2^ values of 0.98 for θ_*ankle*_ and 0.97 for τ_*ankle*_ using support vector regression for level ground walking at self-selected normal speeds. However, the difference in selection of input features, difference in datasets, amount of data used, and different performance quantification measures makes a direct comparison with other studies difficult. Nevertheless, none of these studies have attempted prediction of gait variables for amputee subjects where the impaired gait patterns and incomplete user specific training data poses challenges for using the machine learning regression algorithms for active prosthesis control. We believe that our study is a necessary initial step in this direction.

The proposed random forest approach could be potentially used to devise a high-level control strategy for active ankle prostheses which could predict the ankle angles or moments continuously during level-ground walking at varying speeds. A low-level controller could take the output of the high-level controller as input and directly generate the required moments (for torque control) or angles (for position control) or both (for a composite torque-position control during stance and swing phase, respectively). The input features used in this study could be obtained using wearable sensors like goniometers or IMUs which measure joint or limb rotations in real-time. Since the inputs are required only from the ipsilateral side, the non-prosthetic side does not need to be instrumented.

There were a few limitations to our study. First, the data used in our study was acquired from motion capture experiments. However, for real-life locomotive conditions, the data need to be acquired using wearable sensors which may contain artifacts that were not taken into consideration here. Second, the subject specific models for amputees were trained with data acquired while the amputees walked with a passive prosthesis. Therefore, adaptability of the model to changes in the prosthetic set-up constitutes a crucial part of future studies. Using the predicted angles/moments for controlling the active prosthesis might also in turn alter the input patterns to some extent. Thus, imparting robustness to the control model remains another focus of our future study. This might call for an iterative online training of the model to adapt to new training inputs to enhance model efficiency. Furthermore, future studies should also focus on evaluating the proposed subject-specific model on a larger pool of amputee subjects and on different locomotion modes. Finally, the proposed approach should be validated on online experiments to control an active prosthesis.

## 5. Conclusion

We investigated the feasibility of two different approaches—subject-specific and inter-subject—for training a random forest model using incomplete amputee-specific training data for predicting the ankle angle and ankle moment during level-ground walking. We found that a random forest subject-specific model predicted the required normative ankle motion with up to three times lower errors than an inter-subject model and could generalize its learning to different speeds up to a difference of 0.4 m/s from the speed which it was trained. These results are promising and suggest that the proposed random-forest based model could be used to device a robust control strategy for an intelligent prosthetic ankle, which can adapt to its specific user at varying speeds and produce the required ankle angle or moment similar to an able-bodied walking gait. The general approach could also be useful in other fields with incomplete training data, e.g., other human-computer interfaces like upper limb prosthetics, assistive robotics, e.g., cyborg and bionic technologies.

## Data Availability Statement

The datasets of able-bodied subjects used in this study can be found in Figshare (doi: 10.6084/m9.figshare.5722711). The amputee datasets used are not publicly available due to confidentiality of patient data.

## Ethics Statement

The studies involving human participants were reviewed and approved by Ethics Committee of University Medical Center Göttingen, Göttingen, Germany (application number: 26/3/18). The patients/participants provided their written informed consent to participate in this study.

## Author Contributions

SD conceptualized the study, performed the analyses, and wrote the first draft of the manuscript. TY collected and processed the experimental data and revised the manuscript. AS supervised the clinical experiment, bridged clinical and mathematical approaches, and revised the manuscript. All authors contributed to manuscript revision, read, and approved the submitted version.

## Conflict of Interest

The authors declare that the research was conducted in the absence of any commercial or financial relationships that could be construed as a potential conflict of interest.

## References

[B1] Allard P. Leteneur S. Watelain É. Begon M. (2017). Urban legends in gait analysis. Mov. Sport Sci. Sci. Motric. 98, 5–11. 10.3917/sm.098.0005

[B2] Ardestani M. M. Zhang X. Wang L. Lian Q. Liu Y. He J. (2014). Human lower extremity joint moment prediction: a wavelet neural network approach. Expert Syst. Appl. 41, 4422-4433. 10.1016/j.eswa.2013.11.003

[B3] Au S. Berniker M. Herr H. (2008). Powered ankle-foot prosthesis to assist level-ground and stair-descent gaits. Neural Netw. 21, 654-666. 10.1016/j.neunet.2008.03.006 18499394

[B4] Au S. K. Bonato P. Herr H. (2005). An EMG-position controlled system for an active ankle-foot prosthesis: an initial experimental study, in ICORR 2005. 9th International Conference on Rehabilitation Robotics (Chicago, IL: IEEE), 375–379. 10.1109/ICORR.2005.1501123

[B5] Bartlett H. L. Goldfarb M. (2017). A phase variable approach for imu-based locomotion activity recognition. IEEE Trans. Biomed. Eng. 65, 1330–1338. 10.1109/TBME.2017.2750139 28910754

[B6] Biau G. (2012). Analysis of a random forests model. J. Mach. Learn. Res. 13, 1063–1095. 10.5555/2188385.2343682

[B7] Biau G. Scornet E. (2016). A random forest guided tour. Test 25, 197–227. 10.1007/s11749-016-0481-7

[B8] Boudali A. M. Sinclair P. J. Smith R. Manchester I. R. (2017). Human locomotion analysis: Identifying a dynamic mapping between upper and lower limb joints using the Koopman operator, in 2017 39th Annual International Conference of the IEEE Engineering in Medicine and Biology Society (EMBC) (Seogwipo: IEEE), 1889–1892. 10.1109/EMBC.2017.8037216 29060260

[B9] Breiman L. (2001). Random forests. Mach. Learn. 45, 5–32. 10.1023/A:1010933404324

[B10] Breiman L. Friedman J. Olshen R. Stone C. (1984). Classification and regression trees. Wadsworth Int. Group 37, 237–251.

[B11] Delp S. L. Anderson F. C. Arnold A. S. Loan P. Habib A. John C. T.. (2007). Opensim: open-source software to create and analyze dynamic simulations of movement. IEEE Trans. Biomed. Eng. 54, 1940–1950. 10.1109/TBME.2007.901024 18018689

[B12] Dey S. Eslamy M. Yoshida T. Ernst M. Schmalz T. Schilling A. (2019). A support vector regression approach for continuous prediction of ankle angle and moment during walking: an implication for developing a control strategy for active ankle prostheses, in 2019 IEEE 16th International Conference on Rehabilitation Robotics (ICORR) (Toronto, ON: IEEE), 727–733. 10.1109/ICORR.2019.8779445 31374717

[B13] Douglas P. K. Harris S. Yuille A. Cohen M. S. (2011). Performance comparison of machine learning algorithms and number of independent components used in fmri decoding of belief vs. disbelief. Neuroimage 56, 544–553. 10.1016/j.neuroimage.2010.11.002 21073969 PMC3099263

[B14] Eilenberg M. F. Geyer H. Herr H. (2010). Control of a powered ankle-foot prosthesis based on a neuromuscular model. IEEE Trans. Neural Syst. Rehabil. Eng. 18, 164–173. 10.1109/TNSRE.2009.2039620 20071268

[B15] Eslamy M. Schilling A. F. (2018). A conceptual high level controller to walk with active foot prostheses/orthoses, in 2018 7th IEEE International Conference on Biomedical Robotics and Biomechatronics (Biorob) (Enschede: IEEE), 1224–1229. 10.1109/BIOROB.2018.8487213

[B16] Fukuchi C. A. Fukuchi R. K. Duarte M. (2018). A public dataset of overground and treadmill walking kinematics and kinetics in healthy individuals. PeerJ 6:e4640. 10.7717/peerj.4640 29707431 PMC5922232

[B17] Geurts P. Ernst D. Wehenkel L. (2006). Extremely randomized trees. Mach. Learn. 63, 3–42. 10.1007/s10994-006-6226-1

[B18] Hof A. L. (1996). Scaling gait data to body size. Gait Posture 3, 222–223. 10.1016/0966-6362(95)01057-2 11323225

[B19] Holgate M. A. Sugar T. G. Bohler A. W. (2009). A novel control algorithm for wearable robotics using phase plane invariants, in ICRA'09. IEEE International Conference on Robotics and Automation (Kobe: IEEE), 3845–3850. 10.1109/ROBOT.2009.5152565

[B20] Hsu C.-W. Chang C.-C. Lin C.-J. (2003). A Practical Guide to Support Vector Classification, Department of Computer Science, National Taiwan University, Taiwan.

[B21] Huang H. Zhang F. Hargrove L. J. Dou Z. Rogers D. R. Englehart K. B. (2011). Continuous locomotion-mode identification for prosthetic legs based on neuromuscular-mechanical fusion. IEEE Trans. Biomed. Eng. 58, 2867–2875. 10.1109/TBME.2011.2161671 21768042 PMC3235670

[B22] Hultquist C. Chen G. Zhao K. (2014). A comparison of gaussian process regression, random forests and support vector regression for burn severity assessment in diseased forests. Rem. Sens. Lett. 5, 723–732. 10.1080/2150704X.2014.963733

[B23] Jezernik S. Colombo G. Keller T. Frueh H. Morari M. (2003). Robotic orthosis lokomat: a rehabilitation and research tool. Neuromodulation 6, 108–115. 10.1046/j.1525-1403.2003.03017.x 22150969

[B24] Khah S. S. Wu Y. (2019). An enhanced ad event-prediction method based on feature engineering. arXiv 1907.01959.

[B25] Lawson B. E. Shultz A. H. Goldfarb M. (2013). Evaluation of a coordinated control system for a pair of powered transfemoral prostheses, in 2013 IEEE International Conference on Robotics and Automation (Karlsruhe: IEEE), 3888–3893. 10.1109/ICRA.2013.6631124

[B26] Leardini A. Sawacha Z. Paolini G. Ingrosso S. Nativo R. Benedetti M. G. (2007). A new anatomically based protocol for gait analysis in children. Gait Posture 26, 560–571. 10.1016/j.gaitpost.2006.12.018 17291764

[B27] Little V. L. McGuirk T. E. Patten C. (2013). So-called ‘foot-drop’ post-stroke: not a dorsiflexor impairment, in Converging Clinical and Engineering Research on Neurorehabilitation eds Pons J. L. Torricelli D. Pajaro M. (Toledo: Springer), 691–695. 10.1007/978-3-642-34546-3_112

[B28] Mo S.-M. Hwang J. Kim J. H. Jung M.-C. (2019). The ergonomic design of wearable robot based on the shoulder kinematic analysis by walking speed, in International Conference on Applied Human Factors and Ergonomics (Washington, DC: Springer), 63–69. 10.1007/978-3-030-20476-1_8

[B29] Pinzone O. Schwartz M. H. Baker R. (2016). Comprehensive non-dimensional normalization of gait data. Gait Posture 44, 68–73. 10.1016/j.gaitpost.2015.11.013 27004635

[B30] Quinlan J. R. (1986). Induction of decision trees. Mach. Learn. 1, 81–106. 10.1007/BF00116251

[B31] Quintero D. Villarreal D. J. Gregg R. D. (2016). Preliminary experiments with a unified controller for a powered knee-ankle prosthetic leg across walking speeds, in 2016 IEEE/RSJ International Conference on Intelligent Robots and Systems (IROS) (Daejeon: IEEE), 5427–5433. 10.1109/IROS.2016.7759798 PMC5381823 28392969

[B32] Riley P. O. Paolini G. Della Croce U. Paylo K. W. Kerrigan D. C. (2007). A kinematic and kinetic comparison of overground and treadmill walking in healthy subjects. Gait Posture 26, 17–24. 10.1016/j.gaitpost.2006.07.003 16905322

[B33] Schurr S. A. Marshall A. N. Resch J. E. Saliba S. A. (2017). Two-dimensional video analysis is comparable to 3D motion capture in lower extremity movement assessment. Int. J. Sports Phys. Therapy 12:163. 28515970 PMC5380858

[B34] Senden R. Meijer K. Heyligers I. Savelberg H. Grimm B. (2012). Importance of correcting for individual differences in the clinical diagnosis of gait disorders. Physiotherapy 98, 320-324. 10.1016/j.physio.2011.06.002 23122438

[B35] Silverman A. K. Fey N. P. Portillo A. Walden J. G. Bosker G. Neptune R. R. (2008). Compensatory mechanisms in below-knee amputee gait in response to increasing steady-state walking speeds. Gait Posture 28, 602–609. 10.1016/j.gaitpost.2008.04.005 18514526

[B36] Stansfield B. Hillman S. Hazlewood M. Lawson A. Mann A. Loudon I.. (2003). Normalisation of gait data in children. Gait Posture 17, 81–87. 10.1016/S0966-6362(02)00062-0 12535730

[B37] Sup F. Bohara A. Goldfarb M. (2008). Design and control of a powered transfemoral prosthesis. Int. J. Robot. Res. 27, 263–273. 10.1177/0278364907084588 19898683 PMC2773553

[B38] Tsukahara A. Hasegawa Y. Sankai Y. (2011). Gait support for complete spinal cord injury patient by synchronized leg-swing with HAL, in 2011 IEEE/RSJ International Conference on Intelligent Robots and Systems (San Francisco, CA: IEEE), 1737–1742. 10.1109/IROS.2011.6094827

[B39] Tucker M. R. Olivier J. Pagel A. Bleuler H. Bouri M. Lambercy O.. (2015). Control strategies for active lower extremity prosthetics and orthotics: a review. J. Neuroeng. Rehabil. 12:1. 10.1186/1743-0003-12-1 25557982 PMC4326520

[B40] Varol H. A. Goldfarb M. (2007). Decomposition-based control for a powered knee and ankle transfemoral prosthesis, in 2007 IEEE 10th International Conference on Rehabilitation Robotics (Noordwijk: IEEE), 783–789. 10.1109/ICORR.2007.4428514

[B41] Varol H. A. Sup F. Goldfarb M. (2010). Multiclass real-time intent recognition of a powered lower limb prosthesis. IEEE Trans. Biomed. Eng. 57, 542–551. 10.1109/TBME.2009.2034734 19846361 PMC2829115

[B42] Villarreal D. J. Poonawala H. A. Gregg R. D. (2016). A robust parameterization of human gait patterns across phase-shifting perturbations. IEEE Trans. Neural Syst. Rehabil. Eng. 25, 265–278. 10.1109/TNSRE.2016.2569019 27187967 PMC5107364

[B43] Wahid F. Begg R. Lythgo N. Hass C. J. Halgamuge S. Ackland D. C. (2016). A multiple regression approach to normalization of spatiotemporal gait features. J. Appl. Biomech. 32, 128–139. 10.1123/jab.2015-0035 26426798

[B44] Windrich M. Grimmer M. Christ O. Rinderknecht S. Beckerle P. (2016). Active lower limb prosthetics: a systematic review of design issues and solutions. Biomed. Eng. Online 15:140. 10.1186/s12938-016-0284-9 28105948 PMC5249019

[B45] Winter D. A. Sienko S. E. (1988). Biomechanics of below-knee amputee gait. J. Biomech. 21, 361–367. 10.1016/0021-9290(88)90142-X 3417688

